# Toll-like Receptor Activation Induces Degeneration of Human Intervertebral Discs

**DOI:** 10.1038/s41598-017-17472-1

**Published:** 2017-12-07

**Authors:** Emerson Krock, Derek H. Rosenzweig, J. Brooke Currie, Daniel G. Bisson, Jean A. Ouellet, Lisbet Haglund

**Affiliations:** 1 Orthopaedic Research Lab, Department of Surgery, McGill University and the Research Institute of the McGill University Health Centre, 1650 Cedar Ave, C10-148.8, Montreal, QC, H3G 1A4 Canada; 2 McGill Scoliosis and Spine Group, Department of Surgery, McGill University and the Research Institute of the McGill University Health Centre, 1650 Cedar Ave, C10-148.8, Montreal, QC, H3G 1A4 Canada; 3 Shriner’s Hospital for Children, 1003 Decarie Blvd, Montreal, QC, H4A 0A9 Canada

## Abstract

Toll-like receptors (TLR) are activated by endogenous alarmins such as fragmented extracellular matrix compounds found in the degenerating disc. TLRs regulate cytokine, neurotrophin, and protease expression in human disc cells *in vitro*, and thus control key factors in disc degeneration. However, whether TLR activation leads to degenerative changes in intact human discs is unclear. Nucleus pulposus (NP) cells isolated from non-degenerating discs increase IL-1β and nerve growth factor gene expression following treatment with Pam2CSK4 (TLR2/6 agonist) but not Pam3CSK4 (TLR1/2 agonist). Challenging NP cells with Pam2CSK4 or 30 kDa fibronectin fragments (FN-f, an endogenous TLR2 and TLR4 alarmin) increased secretion of proinflammatory cytokines. We then investigated the effect of TLR activation in intact, non-degenerate, *ex vivo* human discs. Discs were injected with PBS, Pam2CSK4 and FN-f, and cultured for 28 days. TLR activation increased proteoglycan and ECM protein release into the culture media and decreased proteoglycan content in the NP. Proteases, including MMP3, 13 and HTRA1, are secreted at higher levels following TLR activation. In addition, proinflammatory cytokine levels, including IL-6, TNFα and IFNγ, increased following TLR activation. These results indicate that TLR activation induces degeneration in human discs. Therefore, TLRs are potential disease-modifying therapeutic targets to slow disc degeneration.

## Introduction

Chronic low back is a leading cause of disability and morbidity world-wide^1,2^, and intervertebral disc degeneration is one of the main aetiologies^3^. Despite the high prevalence and the associated health care and socioeconomic costs, limited knowledge of disc pathology and pain restrict therapeutic approaches. Currently, no disease modifying drugs exist for this clinical problem. The early stages of disc degeneration are the ideal disease stage for disease modifying interventions that could stop or slow the progression of degeneration^4^. However, the early stages of degeneration in particular are poorly understood.

The disc is a fibrocartilaginous tissue consisting of the central, gelatinous nucleus pulposus (NP), which is surrounded by the annulus fibrosus (AF). The NP is composed primarily of proteoglycans, especially aggrecan, and randomly organized fibrils of type II collagen. As a result, the NP is a highly hydrophilic tissue, with high water content and gel-like characteristics, allowing for resistance to compressive forces on the spine. The AF is composed primarily of type I collagen fibrils organized in a laminar structure. The NP and AF are separated from the vertebral bodies by a thin layer of hyaline cartilage called the cartilaginous endplate^3,5^. In non-degenerating discs there is a balance between ECM synthesis and degradation, resulting in a slow, physiological ECM turnover^5^. During disc degeneration however, ECM turnover is no longer balanced and catabolism outpaces ECM synthesis. The proteoglycans, and other ECM proteins, such as fibronectin, collagens and a number of small leucine-rich repeat proteins (SLRPs), are degraded and fragmented^3,6^. Upregulated proteases, including cathepsins, matrix metalloproteinases MMP-3 and -13, ADAMTS-4 and -5^7,8^ and HTRA1^9,10^, degrade the ECM.

Sterile inflammation, which is an increase of proinflammatory factors in the absence of infection, is another hallmark of disc degeneration. Proinflammatory cytokines, such as IL-1β, TNFα, IL-6 and IL-8, as well as angiogenic factors and neurotrophins all increase^11–17^ during degeneration. Cytokines increase the expression of proteases, neurotrophins, cytokines and cytokine receptors. Cytokines and neurotrophins are also linked to pain^18–21^. The sterile inflammation of the disc creates a feed-forward loop that increases ECM degeneration, leading to disc degeneration. However, cytokines like IL-1β and TNFα are expressed at low to undetectable levels in non-degenerating discs^11,22,23^. Therefore, cytokines alone do not explain the sterile inflammation that occurs during early stages disc degeneration. Increased understanding of sterile inflammation and early stages of disc degeneration could identify new therapeutic targets.

ECM degradation is a key event even in the earliest stages of disc degeneration. ECM fragments can act as danger associated molecular patterns (DAMPs), also termed alarmins. Due to poor waste exchange, matrix fragments also may slowly accumulate during physiological matrix turnover. Alarmins can activate pattern recognition receptors including toll-like receptors (TLRs). TLRs were originally characterized for their role in innate immunity, but more recently alarmins were also found to activate TLRs. Extracellular matrix fragments that act as alarmins include fragmented aggrecan^24^, fibronectin, biglycan, decorin^25^ and low molecular weight hyaluronic acid^26^. Extracellular high mobility group box 1 (HMGB1) also activates TLRs and likely functions as an alarmin in disc degeneration^27^. Human disc cells express TLR1-6, 9 and 10, and TLRs 1, 2, 4 and 6 all increase with the grade of degeneration^28^. Interestingly, cells from non-degenerating discs express TLR1, 2, 4 and 6, and adverse mechanical strain increases the expression of TLR2 and 4^29^. In cartilage and disc tissue TLR2 and 4 are the TLRs primarily associated with alarmin recognition and subsequent activation by alarmins. TLR2 activation in human NP or AF cells activates NF-κB, p38 and ERK signalling, which are associated with disc degeneration^22,30^. Furthermore, TLR2 activation increases NGF, BDNF, IL-1β, TNFα, IL-6, MMP-1-3, and -13 and COX-2 in human disc cells^10,22,28^. TLR4 activation in human NP and AF cells also increases neurotrophin and cytokine gene expression, although inconsistently^22^. These previous studies demonstrate that TLR activation increases key components of disc degeneration (proteases, cytokines and neurotrophins) *in vitro*, which could create a proinflammatory feed-forward loop *in vivo*. TLRs therefore likely play a role in disc degeneration and chronic low back pain. Due to ECM fragment and alarmin turnover during physiological and pathological matrix degradation, TLRs are potentially activated during the early stages of degeneration. However, it remains unclear if TLR activation is sufficient to induce degenerative changes in human discs.

In the current study, we hypothesized that activation of TLR2 will induce degenerative changes in human intervertebral discs. This study investigated the effects injecting specific synthetic TLR2 agonists and 30 kDa fibronectin fragments (a TLR2 and TLR4 agonist) into non-degenerate human discs in an *ex vivo* organ culture system. We found that TLR activation causes degenerative changes in the NP tissue, increases release of specific ECM components, and increases in proteases and sterile inflammation.

## Results

### Pam2CSK4 increases proinflammatory and pronociceptive gene expression

**Figure 1:**
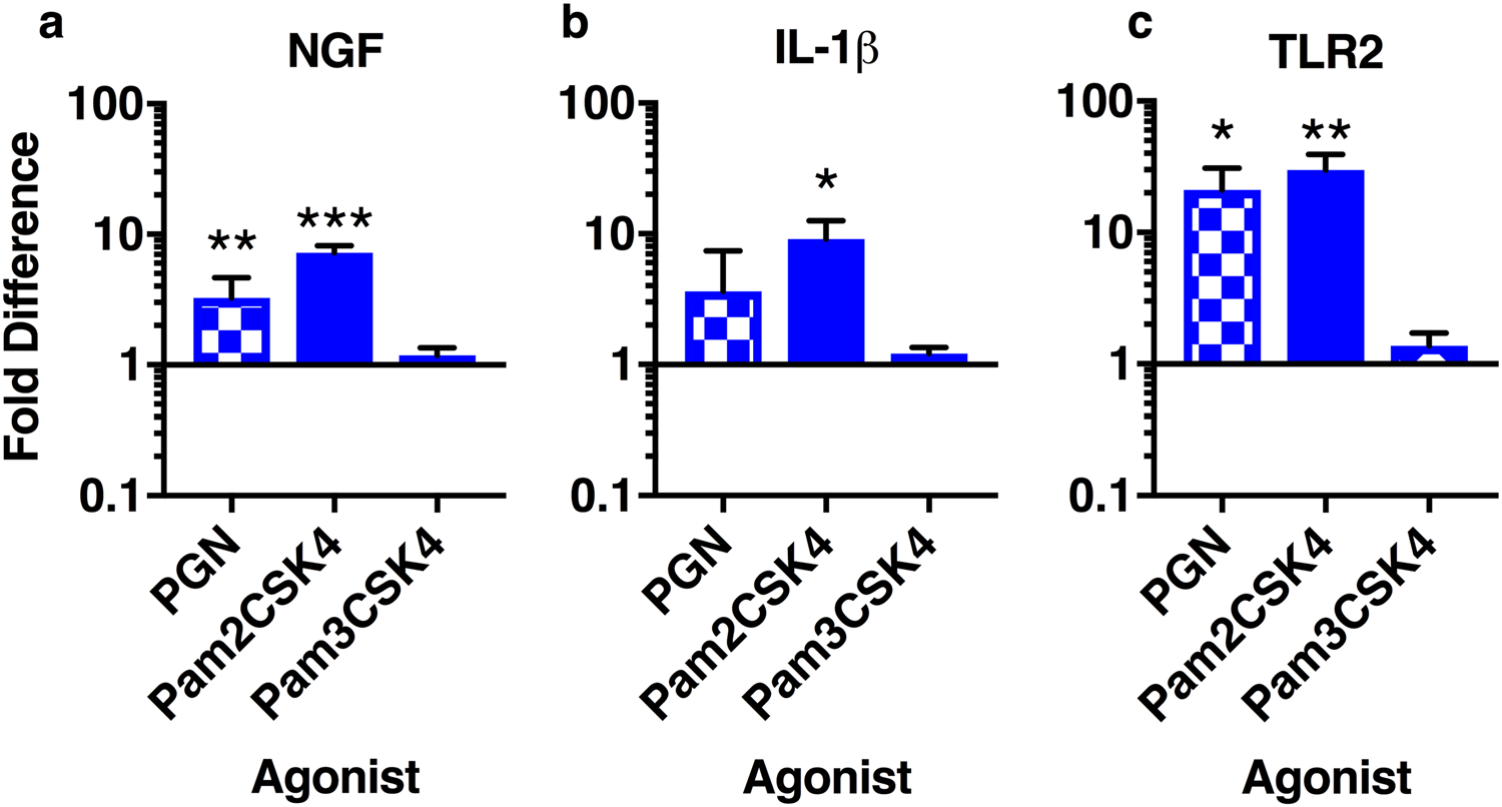
NGF and IL-1β expression following TLR stimulation. NP cells were isolated from non-degenerate human discs and treated with peptidoglycan (PGN, TLR2 agonist), Pam2CSK4 (TLR2/6 and TLR2 agonist) and Pam3CSK4 (TLR1/2 agonist). Nerve growth factor (NGF, **a**), IL-1β (**b**) and TLR2 (**c**) gene expression were evaluated and compared to untreated cells. Data is presented as mean ± SEM and was analyzed by repeated measures one-way ANOVA. *Indicates p < 0.05, **indicates p < 0.01, n = 4.

Previously, we used the non-soluble TLR2 agonist peptidoglycan (PGN) to activate TLR2 and study downstream responses. Agonists likely have to be soluble to have an effect following injection into a disc. We therefore assessed the ability of the soluble TLR2 agonists Pam3CSK4, a TLR2/1 agonist (EC_50_ 0.47–20 ng/ml^31,32^), and Pam2CSK4, a TLR2/6 agonist (EC_50_ 0.015–1 ng/ml^31,32^), to induce inflammatory and nociceptive gene expression. Use of specific agonists will also give insight into which TLR2 heterodimers are more readily activated in human discs. As previously reported^22^, PGN increases NGF, IL-1β, and TLR2 expression in NP cells compared to untreated cells (Fig. 1). Pam3CSK4 had no effect on NGF, IL-1β or TLR2 expression whereas Pam2CSK4 increased NGF (7.2 ± 0.9-fold, Fig. 1a), IL-1β (9.1 ± 3.4, Fig. 1b) and TLR2 (29.9 ± 9.3, Fig. 1c) expression.

### Fibronectin fragments activate TLR2

To determine maximal effective dosages for Pam2CSK4 and the 30 kDa fibronectin fragments (FN-f), we first challenged HEK 293 cells that over expressed TLR2 and NF-κB inducible secreted embryonic alkaline phosphatase (SEAP). SEAP detection media turns blue following TLR2 activation and the absorbance can be monitored. Pam2CSK4 concentrations ranged from 0.0002 pM to 15.75 pM and FN-f concentrations ranged from 100 nM to 1600 nM. Both Pam2CSK4 and FN-f increased TLR2 activity in a dose-dependent manner (Supplemental Fig. 1). On a μmolar basis, Pam2CSK4 was more potent than FN-f. Maximal activity, indicated by a plateau of absorbance readings as is the case with Pam2CSK4, was not observed with FN-f. We were unable to test high concentrations of FN-f due to lack of solubility at high concentrations. Regardless, both Pam2CSK4 and FN-f increased TLR2 activity.

### *In vitro* TLR activation increases proinflammatory cytokine production

**Figure 2:**
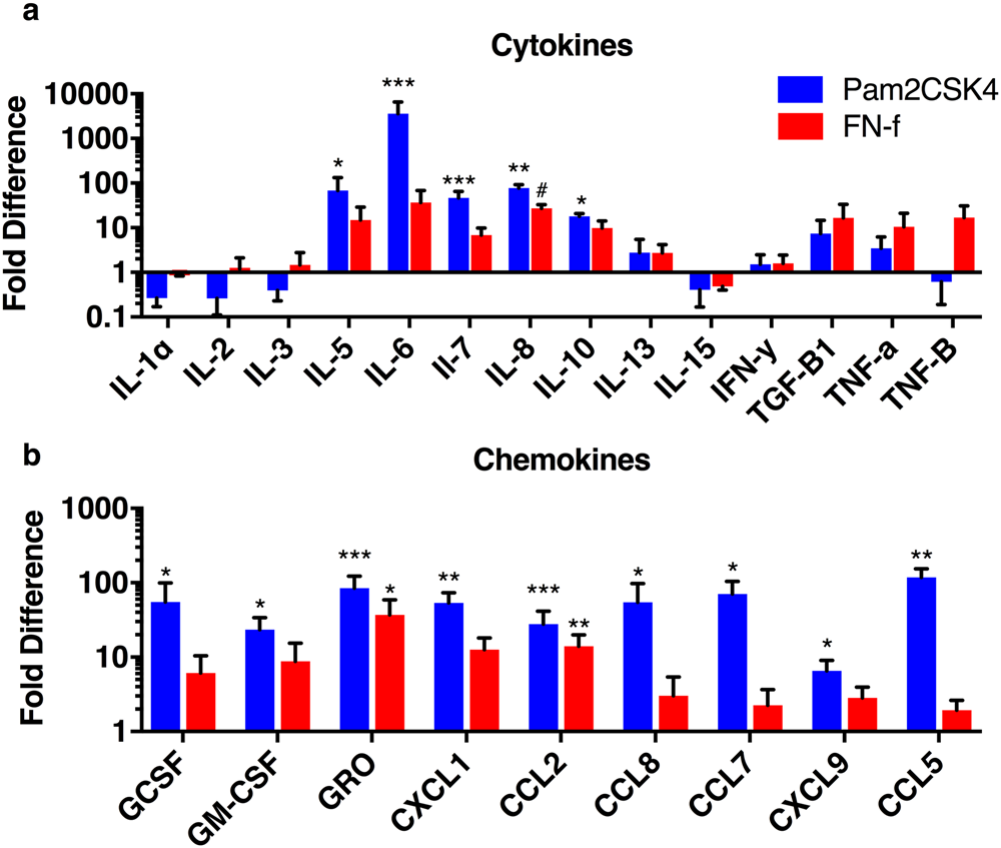
The *in vitro* effect of NP cell TLR activation on cytokine secretion. NP cells were treated with PBS, 100 ng/ml Pam2CSK or 1 μmol 30 kDa fibronectin fragments (FN-f) for 48 hours and conditioned culture media was evaluate using protein arrays. (**a**) Cytokine and (**b**) chemokine levels were normalized to PBS treated cells from the same experiments. Data is presented as the mean ± SEM of the fold difference and analyzed by repeated measures one-way ANOVA. ^#^Indicates p < 0.1, *indicates p < 0.05, **indicates p < 0.01, n = 3.

Nucleus pulposus cells isolated from non-degenerating discs were challenged with 100 ng/ml Pam2CSK or 1 μM FN-f. The Pam2CSK4 dosage was chosen to ensure a maximal response based on Supplemental Fig. 1, whereas the dose of FN-f was chosen to be within the range of fibronectin found in discs^33^. TLR2/6 activation with Pam2CSK4 strongly increases secretion of several proinflammatory cytokines and chemokines such as IL-6, IL-8, CXCL1, GRO and CCL2. FN-f also strongly increases GRO and CCL2, moderately increases cytokines such as IL-8, as well as a clear trend for increasing other factors (Fig. 2a and b). As expected the high dose of Pam2CSK4 gave a stronger and a more robust increase in inflammatory factors than the lower concentration of FN-f.

### TLR agonists induce proteoglycan loss

**Figure 3:**
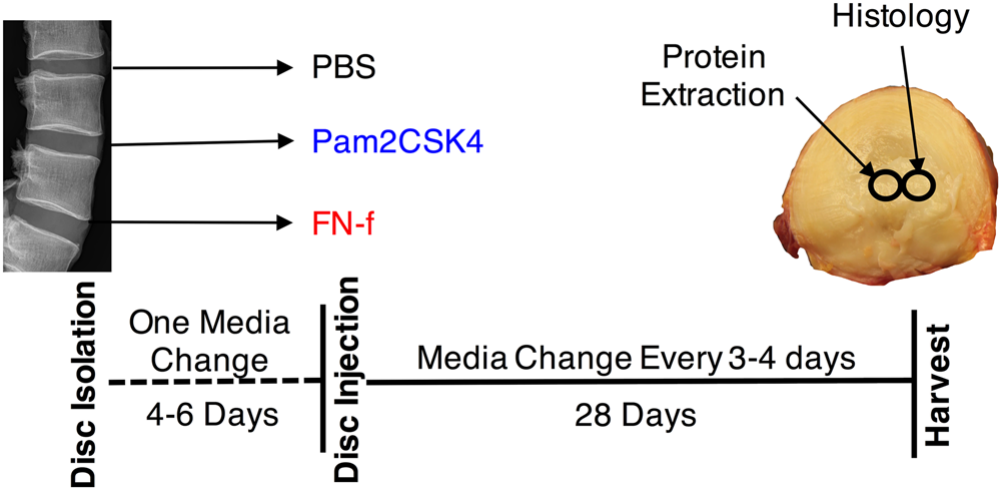
Schematic of the *ex vivo* organ culture experiment. Lumbar spines from organ donors were assessed radiographically for signs of degeneration. Three discs per experiment lacking signs of degeneration were isolated from a spine, cultured for 4–6 days and injected with PBS, Pam2CSK4 of 30 kDa fibronectin fragments. Discs were then cultured for 28 days, with media changes every 3–4 days. NP tissues punches were taken at the end of the experiment.

To determine if TLR activation induces degenerative changes in human discs, lumbar discs lacking signs of degeneration (radiographic and visual morphological examination, grade 0-1 based on Wilke *et al*.^34^, see Supplemental Table 1) were excised from human spinal segments and cultured^35^. Following 4–6 days of *ex vivo* whole disc organ culture, discs were injected with PBS (200–250 μl), Pam2CSK4, (100 ng/g disc tissue in 200–250 μl PBS) or FN-f (100 nM/g disc tissue in 200–250 μl PBS), delivered to the centre of the NP. The experimental setup is outlined in Fig. 3.

**Figure 4:**
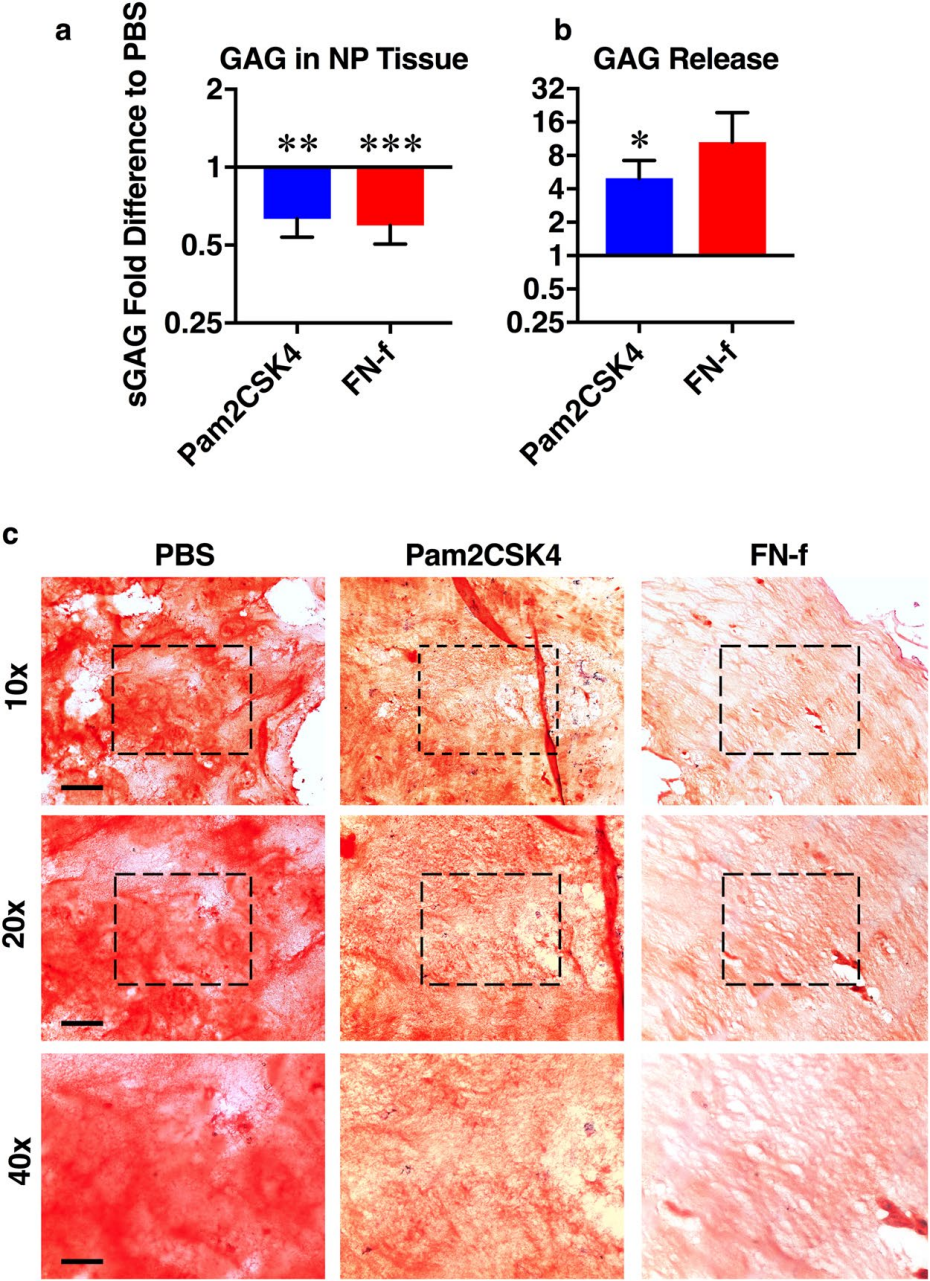
Effect of TLR activation on NP matrix. Sulfated glycosaminoglycan (sGAG) levels in the NP were assessed by DMMB and normalized to discs injected with PBS from the same spine (**a**). Conditioned culture media was pooled from days 4 to 28 and GAG release was assessed by DMMB and normalized to day 0 GAG concentration and then to the sGAG concentration of PBS injected disc media from the same spine (**b**). Histological sections were stained with safranin-O and imaged with 10×, 20×, and 40× objectives (**c**). Representative images are shown. Scale bars represent 200, 100, 50 μm under 10×, 20×, and 40× magnification respectively. Data is presented as mean fold difference ± SEM and was analyzed by repeated measures one-way ANOVA. *Indicates p < 0.05, **indicates p < 0.01, n = 6.

After 28-days, sulphated glycosaminoglycan (sGAG) content was assessed by DMMB assay. Pam2CSK4 and FN-f injection reduced sGAG content in the NP tissue compared to discs injected with PBS from the same spine (1.6 ± 0.1 fold-decrease and 1.7 ± 0.09 fold-decrease Fig. 4a). Conditioned media from day 4 through day 28 was pooled and also assessed by DMMB. Compared to PBS injected discs, Pam2CSK4 significantly increased sGAG release into media (4.99 ± 2.2 fold-increase). FN-f also increased sGAG release into the media (10.5 ± 8.9 fold-increase), but the response was more variable, and therefore didn’t reach statistical significance (Fig. 4b).

We further assessed NP ECM degeneration following TLR activation with safranin-O staining. Safranin-O binds negatively charged molecules, which are primarily represented by proteoglycan in the NP. Twenty-eight days after Pam2CSK4 and FN-f injection, tissue biopsies from the NP were taken and histological sections were stained. Both Pam2CSK4 and FN-f decrease safranin-O stain compared to PBS injected discs, further suggesting that TLR activation leads to a loss of proteoglycan (Fig. 4c). Taken together, these results indicate that Pam2CSK4 and FN-f injection decrease proteoglycan content in the NP, suggesting the TLR activation results in NP matrix degeneration.

### TLR activation promotes release of ECM proteins

**Figure 5:**
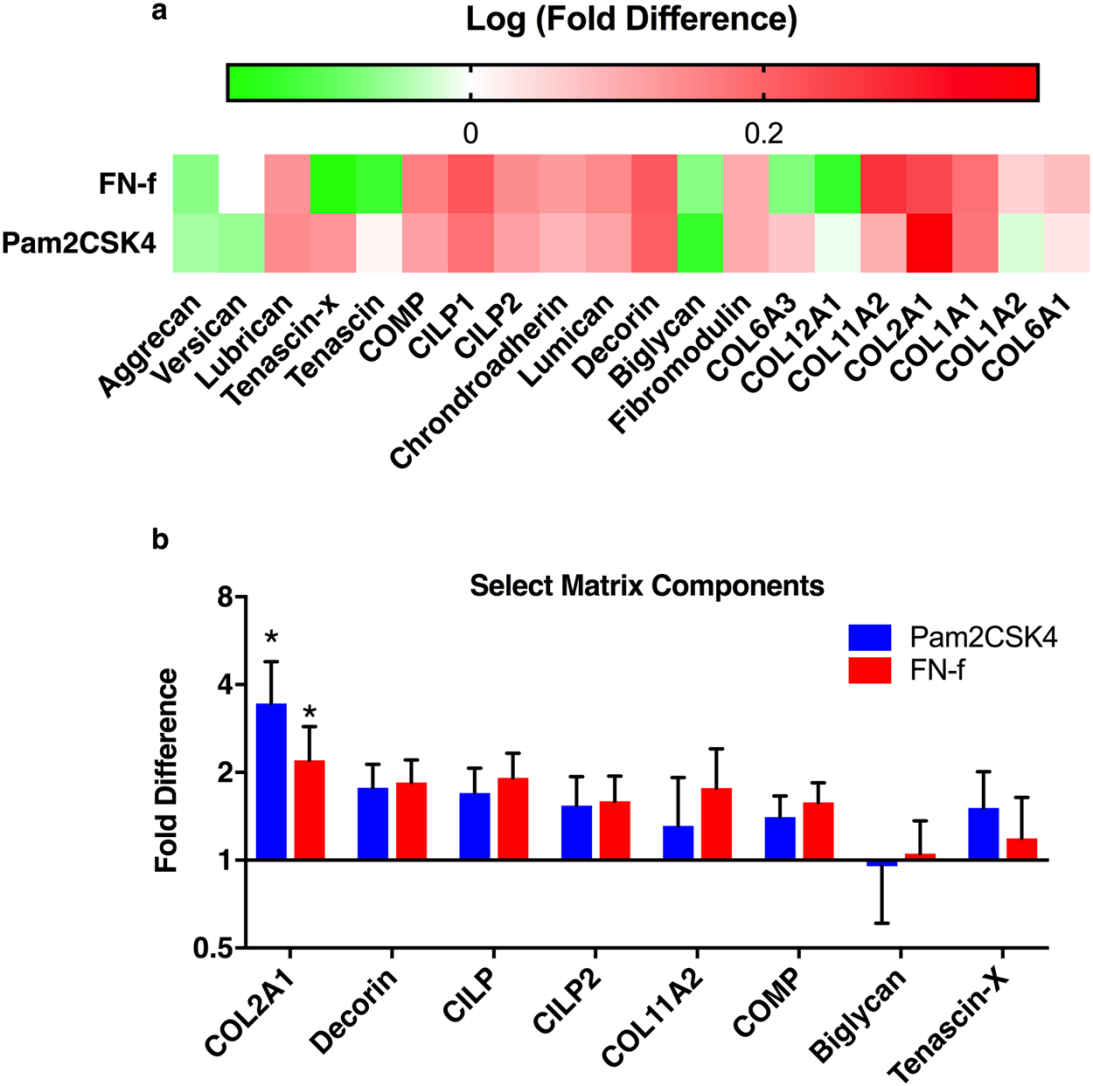
Release of matrix components following TLR activation. Conditioned culture media was pooled from days 4 to 28 and analyzed by mass spectrometry. Spectral counts were normalized to PBS injected discs from the same spine. (**a**) Data is presented as the log of the fold difference. (**b**) Select matrix components are shown as mean fold difference ± SEM. Data was analyzed by repeated measures one-way ANOVA. *Indicates p < 0.05, n = 6.

Pooled conditioned media was assessed by mass spectrometry to evaluate the effect of TLR activation on the release of disc ECM components. Compared to PBS injected discs, Pam2CSK4 and FN-f injections increased the release of matrix scaffolding proteins, small leucine-rich repeat proteins, and a number of collagen sub-types as indicated by increased red on the heat map (Fig. 5a). Release of the proteoglycans aggrecan and versican did not increase, although lubrican did increase. Pam2CSK4 significantly increases type II collagen release (3.4 ± 1.4). Decorin, CILP and COMP were also found in higher amounts (Fig. 5b). Taken together, Pam2CSK4 and FN-f injection increased ECM component release indicating that TLR activation leads to overall degradation of the ECM.

### TLR agonists increase protease levels

**Figure 6:**
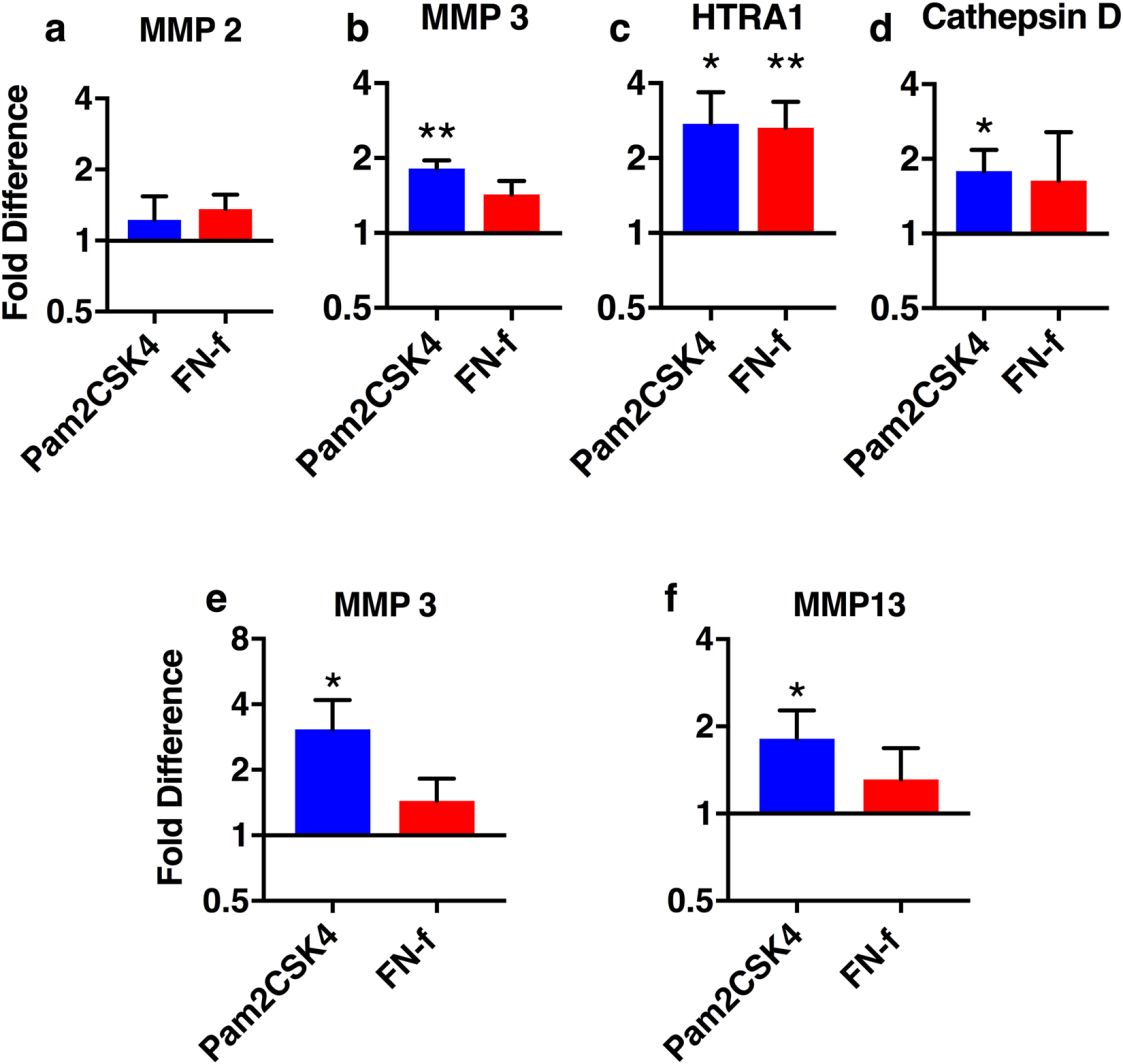
The effect of TLR activation on protease secretion. Conditioned media was pooled from days 4 through 28 and analyzed with mass spectrometry (**a**–**d**) and ELISA (**e**,**f**). Mass spectrometry data was analyzed using spectral counting. All data was normalized to PBS injected discs from the same spine and is presented as the mean fold difference ± SEM. Data was analyzed by repeated measures one-way ANOVA. *Indicates p < 0.05, **indicates p < 0.01, n = 6.

TLR activation in most cell types results in increased production of matrix degrading enzymes, and we therefore evaluated protease abundance in Pam2CSK and FN-f treated-discs. Conditioned media from day 4 through day 28 was pooled and analyzed by mass spectrometry. A number of proteases were detected in conditioned culture media, including several with known roles in disc degeneration. MMP2 slightly increased following injection of both agonists (Fig. 6a). Pam2CSK4 clearly increased the expression of MMP 3 (1.82 ± 0.1, Fig. 6b), HTRA1 (2.75 ± 0.9, Fig. 6c) and Cathepsin D (1.78 ± 0.3, Fig. 6d). FN-f also clearly increased HTRA1 (2.65 ± 0.7, Fig. 6c) secretion and to a lesser extent MMP 3 (Fig. 6b) and Cathepsin D (Fig. 6d).

Along with MMP3, MMP13 is one of the main proteases described in disc degeneration^18,20^, but it was not detected by mass spectrometry. To further evaluate if TLR activation could induce MMP13 secretion and to quantify levels of MMP3, ELISAs were used. Confirming the mass spectrometry data, MMP3 secretion is increased by Pam2CSK4 injection (1.64 ± 0.4-fold increase, 45.2 ± 9.6 ng/ml) and FN-f (1.49 ± 0.31 fold-increase, 49.66 ± 11.6 ng/ml) compared to PBS (31.4 ± 7.2 ng/ml, Fig. 6e). Pam2CSK4 injection also increased MMP13 secretion (1.82 ± 0.5 fold-increase, 3.7 ± 0.69 ng/ml) compared to PBS injected discs (2.3 ± 0.55 ng/ml, Fig. 6f). The MMP13 concentrations measured by ELISA are an order of magnitude lower than MMP3, possibly explaining why MMP13 was not detected by mass spectrometry. Together, mass spectrometry and ELISA data show that TLR activation in intact IVDs results in increased levels of proteases that could degrade the disc ECM.

### TLR activation induces a sterile inflammatory response

**Figure 7:**
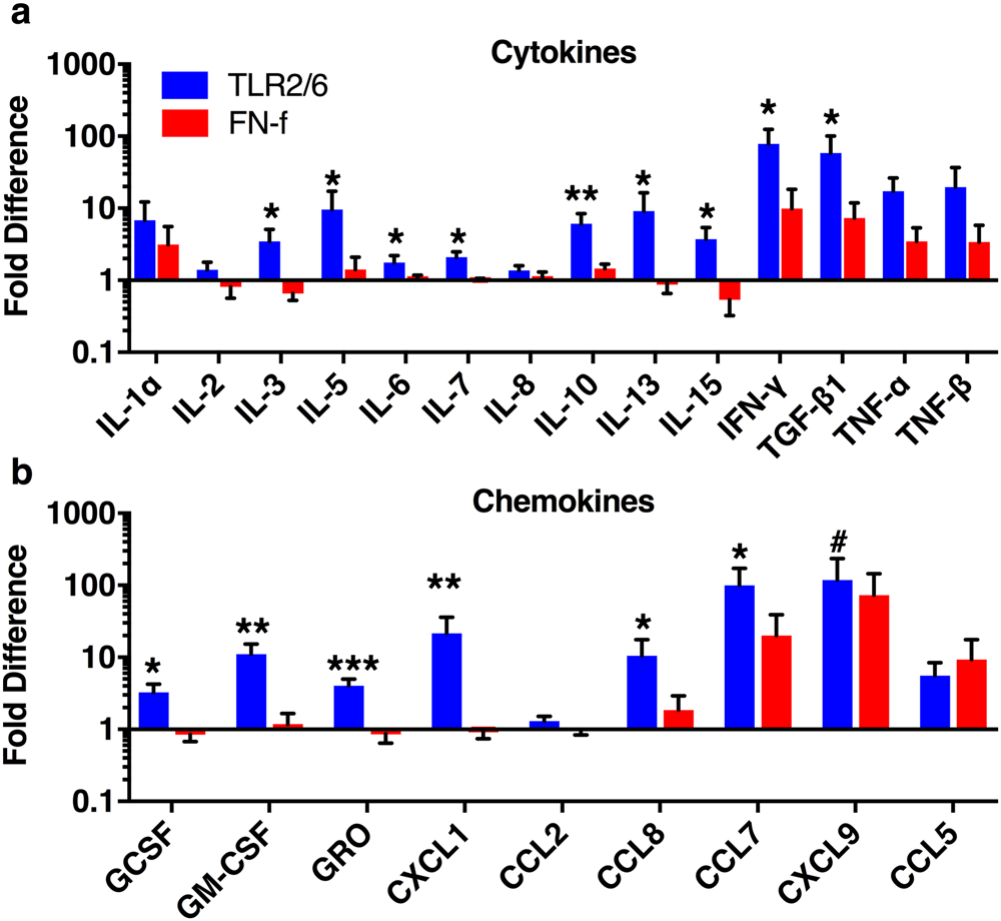
Inflammatory factor secretion following TLR activation. Conditioned media was pooled from days 4 through 28 and analyzed for inflammatory factors. Cytokines (**a**) and chemokines (**b**) were analyzed by protein arrays. Data was normalized to PBS injected discs from the same spine and are show as the mean fold difference ± SEM. Data was analyzed by repeated measures one-way ANOVA. ^#^Indicates p < 0.1, *indicates p < 0.05, **indicates p < 0.01, n = 6.

In addition to increased matrix degradation and protease production, sterile inflammation is another hallmark of disc degeneration^20^. In fact, *ex vivo* degenerating discs from chronic low back pain patients secrete increased levels of many proinflammatory cytokines^11^. To evaluate whether TLR activation by Pam2CSK4 or FN-f increases cytokine secretion, conditioned media from day 4 through day 28 was pooled and assessed by antibody protein arrays. Compared to PBS injected discs, Pam2CSK4 robustly increased secretion of many cytokines (Fig. 7a) and chemokines (Fig. 7b) including IFN-γ (78.4 ± 46.3 fold-increase), GM-CSF (11.1 ± 4.3) and CXCL1 (21.6 ± 14.6) and to a lesser extent increased others including IL-1α, TNFα and CCL8. FN-f injection at this concentration showed a less pronounced increase in cytokine release, although it robustly increased CXCL9 (73.2 ± 70.4) secretion and to a lesser extent increased IL-1α, IFNγ, and TNFα. Sterile inflammation often results in activation of the complement system^36^. Mass spectrometry analysis of pooled conditioned media showed that Pam2CSK4 and FN-f injection moderately increased a number of components of the complement system (data not shown). These results further support that activation of TLRs in human discs induces sterile inflammation leading to IVD degeneration.

## Discussion

**Figure 8:**
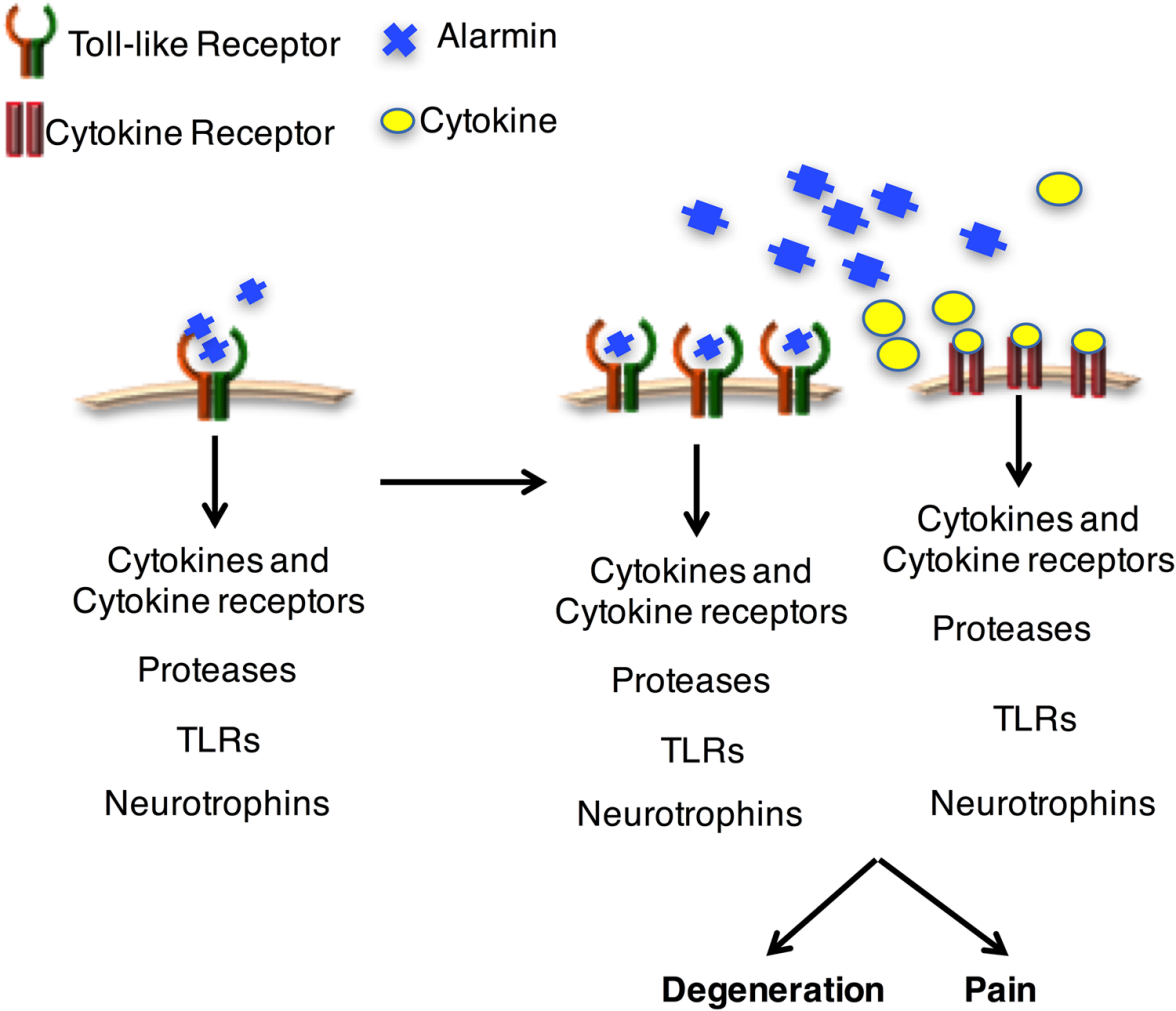
Schematic of a TLR regulated feed-forward degeneration cycle. Early activation of TLRs leads to initial increases in cytokines, cytokine receptors, TLRs, neurotrophins and proteases. In turn, this creates more alarms, leading to enhanced TLR activation. Cytokine receptor activation increases cytokines, cytokine receptors, TLRs, neurotrophins and proteases as well, creating a viscous feed-forward degeneration cycle that results in disc degeneration and pain.

Despite disc degeneration and chronic low back pain being a leading cause of morbidity, no disease modifying drugs exist. This is partly due to the poor understanding of the early stages of disc degeneration. Decreased matrix synthesis, ECM degradation, increased proteases and sterile inflammation are hallmarks of disc degeneration^20^, but how physiological matrix turnover and disc aging transitions to pathological characteristics is poorly understood. Here, we investigated if TLR activation leads to degenerative changes in intact, non-degenerate human discs. We found that injection of Pam2CSK4 and 30 kDa fibronectin fragments, an endogenous alarmin found in degenerating discs, decreases proteoglycan content in the NP, increases general matrix degradation, increases protease secretion and results in sterile inflammation. Thus, TLR activation can induce the pathological hallmarks and drive the feed-forward loop that results in disc degeneration as depicted in Fig. 8.

Proteoglycans, especially aggrecan, make up the bulk of the NP, as illustrated by a proteoglycan to collagen ratio of 27:1 in healthy NP tissue^37^. This ECM composition confers a high anionic charge to the NP, resulting in high water content and the NP’s gel like characteristics. Loss of proteoglycan is an early event in disc degeneration leading to loss of biomechanical function. Mass spectrometry did not reveal increased proteoglycan loss likely because aggrecan and versican are highly glycosylated making detection by mass spectrometry difficult. The glycosylated parts of aggrecan are the first regions lost from degrading cartilaginous tissues. They are rapidly lost from articular cartilage and lost at a much slower rate from discs. However the non-glycosylated fragments are retained in the ECM as is evident by the accumulation of aggrecan G1 region in the tissue^5,8,38,39^. Here we used DMMB and Safrainin-O staining to illustrate that TLR activation decreases proteoglycan content in the NP and increases sGAG release into condition media. These results show that anionic sGAG-chains responsible for water retention are lost following TLR activation. The speed of sGAG loss is likely increased in this culture system compared to *in vivo* loss, but demonstrates a potential mechanism regarless.

Release of several specific ECM components further supports the conclusion that TLR activation leads to degenerative changes. Type II collagen is the primary collagen in the NP, and its decrease is a hallmark of disc degeneration^3^, which is modelled by TLR activation. A number of ECM proteins involved in matrix stability and organization also are released following TLR activation. Cartilage oligomeric matrix protein (COMP) is involved in collagen fibrillogenesis and links a number of other matrix proteins. Furthermore, it correlates with osteoarthritis severity and may have a similar function in disc degeneration^40^. Similarly, cartilage intermediate layer protein (CILP) is another ECM scaffolding protein, and its increased cleavage is associated with osteoarthritis and disc degeneration^40^. SLRPs are also involved in matrix stability, collagen fibril formation and cell-ECM interactions, and SLRP degradation is another specific characteristic of disc degeneration^40,41^. For example, chondroadherin fragmentation generated by HTRA1 at a specific site is a marker of disc degeneration^42^. Taken together, our data indicate that TLR activation leads to cleavage and release of specific matrix components from the disc. This conclusion is further supported by the global decreases in proteoglycan content.

Proteases increase following TLR activation, providing a possible mechanism to explain the increase in matrix components found in the culture media. MMP3 cleaves proteoglycans, such as aggrecan, and collagens and MMP13 cleaves primarily fibrillar collagen^43^. Cathepsin D also cleaves aggrecan and a number of other matrix proteins. HTRA1 cleaves a number of important disc ECM components^44–46^. For example, HTRA1 produces specific chrondroadherin fragments in degenerating discs^42^, it cleaves fibronectin in disc tissue and increases cytokine gene expression and MMP gene expression in intervertebral disc cells^10^. These proteases degrade key components of the disc ECM, including many of those detected in the media by mass spectrometry. Increased protease activity also explains decreased proteoglycan content in the NP following TLR activation. Furthermore, these proteases cleave the ECM to produce fragments that may act as alarmins, including decorin, aggrecan and fibromodulin. Therefore, proteases upregulated by TLRs likely contribute to a feed-forward mechanism resulting in disc degeneration by degrading the ECM and creating new alarmins.

Sterile inflammation is a key characteristic of disc degeneration defined by increases in proinflammatory cytokines and chemokines^47^. *In vitro* human disc cell studies have found TLR2 activation increases IL-1β, TNFα, IL-6, and COX-2. Here, we further show *in vitro* TLR2 activation on human NP cells increases a number of other proinflammatory factors, including IL-8, IL-10, CXCL1 and CCL2. However, whether TLR activation increases proinflammatory factors in intact human discs remained unknown. Here, we show a robust increase in proinflammatory cytokines and chemokines following TLR activation. Furthermore, increased components of the complement system were detected in media from discs, suggesting that the complement system is also activated following TLR stimulation in human discs. The complement system is activated downstream of TLR activation and has roles in regulating inflammatory cytokines in osteoarthritis and rheumatoid arthritis^36,48^, and could have a role in disc degeneration. Cytokines, such as IL-1β, increase cell-surface cytokine receptors, TLRs, and other cytokines and chemokines, further driving the feed-forward cycle of sterile inflammation and disc degeneration^22,28,30^, Furthermore, cytokines also directly increase ECM proteases, such as MMPs and decrease ECM synthesis^17,20,49,50^. TLR regulated sterile inflammation therefore likely contributes to the pathogenesis of disc degeneration.

Interestingly NP cells *in vitro* and intact discs *ex vivo* secrete slightly different cytokine profiles in response to TLR agonists. This could in part be due to the different contexts in which the cells are found between two different experiments. *In vitro* cells cultured in 2D-monolayer are going to receive different environmental cues and be surrounded by different, and less, ECM than disc cells cultured in 3D intact human discs that contain native ECM and different molecular cues. Cell density is also likely higher *in vitro* than in *ex vivo* discs. The *ex vivo* experiments were also much longer (28 days) compared to the *in vitro* experiments (48 hours). These differences in conditions may contribute to the similar but slightly different proinflammatory factor secretion profiles. These differences do however highlight the importance of considering the context and local cellular environment when interpreting results. Similarly, to the differences between *in vitro* and *ex vivo* cytokine production, Pam2CSK4 treatment of disc cells causes a larger increase in proinflammatory cytokines than FN-f. Pam2CSK4 treatment likely results in a greater proinflammatory response because it is a synthetic ligand designed to activate TLR2. FN-f is a naturally occurring ligand and thus likely has different affinities for TLR2. FN-f also contains sites to bind other matrix proteins, such as gelatin binding sites, and therefore will likely bind a number of proteins in addition to TLR2.

The results from the current study clearly demonstrate that TLR activation induces degenerative changes in intact human discs resembling early degenerative changes. The changes in matrix composition and increases in proteases and sterile inflammation suggest a complex feed-forward loop downstream of TLR activation. Therefore, TLR activation may represent an early event in disc degeneration. Breaking this loop may slow disc degeneration and reduce inflammation. Currently, a number of tissue engineering strategies are being investigated to repair discs, but it is likely that progression of degeneration needs to be slowed and sterile inflammation reduced for these strategies to succeed^47^. Furthermore, TLR2 regulates nerve growth factor and brain-derived neurotrophic factor, two potent nociceptive factors found in disc degeneration^22^. We have also recently found that TLR inhibition decreases proinflammatory cytokine secretion and pain-like behaviour in a mouse model of disc degeneration and chronic back pain (Krock *et al*., in revision). Taken together, TLRs are potential disease-modifying therapeutic targets that could slow disc degeneration and reduce chronic low back pain.

## Methods

### Tissue Collection

All procedures were approved by the institutional review board of McGill University (IRB#s A04-M53-08B and A10-M113-13B) for the projects titled “Human Intervertebral Discs used for Culture and Extracellular Matrix” and “Spinal Tissue Repository and Databank Protocol”. All methods were performed in accordance with institutional guidelines and regulations. Lumbar spinal segments (T12-S1) were removed from human organ donors following familial consent. Spines were harvested within 4 hours of aortic cross clamping. Discs lacking radiographic and visual signs of degeneration were used.

### *In vitro* Disc Cell Culture

NP tissues were separated from the AF, mechanically diced and digested overnight with 1.5 mg/ml Collagenase Type II (Gibco). Cells were then cultured and passaged in Dulbecco’s Modified Eagle Medium (DMEM, Sigma-Aldrich) containing 4.5 g/L glucose, 1x Glutamax (Thermo Fisher Scientific), and 50 μg/ml gentamicin (Thermo Fisher Scientific) and 10% fetal bovine serum (FBS, Thermo Fisher Scientific). All experiments were performed in passage 1 or passage 2. Prior to treatment cells were serum starved in DMEM containing 4.5 g/L gluclose, 5 μg/ml Glutamax, 50 μg/ml Gentamicin and 1X insulin transferrin selenium (Thermo Fisher Scientific) for 2 hours. For the experiment examining NP cell responses to difference TLR2 agonists cells were treated in the same serum free media with 5 μg/ml PGN (general TLR2 agonist, Sigma-Aldrich), 100 ng/ml Pam3CSK4 (TLR1/2 specific agonist, Invivogen) or 100 ng/ml Pam2CSK4 (TLR2/6 specific agonist, Invivogen) for 6 hours. The reported EC_50_ for Pam3CSK4 for TLR1/2 ranges from 0.47–20 ng/ml compared to >100 ng/ml for TLR2/6 and the reported EC_50_ for Pam2CSK4 is ranges from 0.015–1 ng/ml compared to approximately 5 ng/ml for TLR1/2^31,32^. RNA was then collected in TRIzol reagent (Thermo Fisher Scientific). For the experiments investigating proinflammatory cytokine secretion, NP cells were serum starved and then treated with 100 ng/ml Pam2CSK5, 1 μM FN-f or vehicle for 48 hours. Conditioned media was collected for analysis by protein array.

### Quantitative PCR

RNA was extracted using the TRIzol chloroform extraction method according to manufacturer’s instructions (Thermo Fisher Scientific). 500 ng of RNA was reverse transcribed using a qScript CDNA Synthesis Kit (Quanta Biosciences) with an Applied Biosystems Verti Thermocycler (Thermo Fisher Scientific). Real-time quantitative PCR was performed using an Applied Biosystems StepOnePlus machine (Thermo Fisher Scientific) with PerfecCTa SYBR Green Fast Mix (Quanta Biosciences). Primers (Thermo Fisher Scientific) are described in Supplemental Table 2. Data was analyzed using the 2^−ΔΔCT^ method. Gene expression was normalized to GAPDH and then to vehicle treated cells.

### HEK 293 cell TLR2 Activity Assay

HEK 293 that are co-transfected with TLR2 and NF-κB inducible SEAP (HEK-Blue hTLR2 reporter cell line [Invivogen, hkb-htlr2]) secrete SEAP in response to TLR2 activation. SEAP then hydrolyzes the colorimetric substrate in the HEK-Blue Detection Media (Invivogen, hb-det2). The optical density (OD) was measured at 630 nm on a ELx808 microplate reader (BioTek) after 12 h of incubation with increasing concentrations of Pam2CSK4 (0.002–20 ng/ml) and FN-f (0.1–1.6 uM).

### *Ex Vivo* Organ Culture and Disc Injection

Three human intervertebral discs from the same spine (n = 7 spines, 21 discs) were isolated and cultured as previously described^9^. Disc characteristics are described in Supplemental Table 1. Discs were evaluated based on Wilke *et al*.^34^ and discs with a grade of 0–1 (on a 0–3 scale) were included for this study. Disc height was determined by averaging the dorsal, ventral and midsection disc height. Briefly, discs were excised from the spine, boney endplates were removed with a high-speed bone burr and cartilaginous endplates were left on the disc. Discs were washed once in PBS and twice in Hanks Balanced Salt Solution (Sigma-Aldrich), both supplemented with 50 μg/ml gentamicin and 0.125 μg/ml Fungizone (Gibco). Discs were then cultured unloaded in DMEM containing 4.5 g/L of glucose supplemented with 5% FBS, 1X Glutamax, 50 μg/ml gentamicin and 50 μg/ml ascorbic acid. After 4–6 days and at least one media change discs from the same spine were injected with PBS, Pam2CSK4 (100 ng/g disc tissue) or 30 kDa FN-f (100 nM/g disc tissue) in a total volume of 200 μl PBS. Discs were then cultured in DMEM supplemented with 1x glutamax, 50 μg/ml gentamicin and 1% FBS for 28 days. Media was changed and collected every 3–4 days. On day 28 tissue punches were taken from the NP for analysis. The experimental setup is depicted in Fig. 3.

### Protein Extraction

Protein from tissue punches was extracted with 4 M GuHCl on a wet-weight per volume basis using 9 volumes extraction buffer (4 M guanidinium hydrochloride, 50 mM sodium acetate, pH 5.8, 10 mM EDTA (all chemicals from Sigma-Alrdich), and COMPLETE® protease inhibitors [Roche, Laval, QC]). Samples were incubated for at least 72 hours at 4 °C with gentle rocking.

### DMMB

Dimethyl methylene blue (DMMB) assays were used according to Mort and Roughley^51^ to quantify sulfated glycosaminoglycans. Chondroitin sulfate (Sigma-Aldrich) was used to make the standard curve and 4 M GuHCl was added to standard curves when quantify tissue sGAG content. All samples were diluted to fall in the middle of the linear portion of the standard curve. Media samples were pooled from days 4 to 28 prior to GAG measurement. sGAG release in media was normalized to the sGAG in media at Day 0 and then normalized to PBS injected discs from the same spine. NP sGAG content was normalized to PBS injected discs from the same spine.

### Histological Staining

Tissue punches (4 mm) were fixed and stored in 80% methanol at 4 °C. Tissues were cyroprotected in 10% (1 h), then 20% (6 h), and then 30% (overnight) sucrose. Samples were embedded in optimal cutting temperature medium (OCT, Thermo Fisher), sectioned at 14 µm and thaw mounted on glass slides. Samples were then stained with Safranin-O (Sigma-Aldrich). Sections were imaged using a Zeiss Axioskop 40 and images were taken with an AxioCam MR (Zeiss), and processed using AxioVision LE64 software (Zeiss).

### Mass Spectrometry

Proteins in media samples were reduced with DTT, alkylated with iodoacetic acid and then digested with trypsin with re-solubilization in 0.1% aqueous formic acid/2% acetonitrile. The peptides were loaded onto a Thermo Acclaim Pepmap (Thermo, 75 uM ID × 2 cm C18 3 uM beads) precolumn and then onto an Acclaim Pepmap Easyspray (Thermo, 75 uM × 15 cm with 2 uM C18 beads) analytical column separation using a Dionex Ultimate 3000 uHPLC at 220 nl/min with a gradient of 2–35% organic (0.1% formic acid in acetonitrile) over 3 hours. Peptides were analyzed using a Thermo Orbitrap Fusion mass spectrometer operating at 120,000 resolution (FWHM in MS1, 15,000 for MS/MS) with HCD sequencing all peptides with a charge of 2+ or greater. The raw data were converted into *.mgf format (Mascot generic format) and searched using Mascot 2.3 against human sequences (Swissprot). The database search results were loaded onto Scaffold Q+ Scaffold_4.4.8 (Proteome Sciences) for spectral counting and data visualization.

### ELISA

MMP3 and MMP13 enzyme linked immunosorbent assays (ELISA) were performed according to manufactures instructions (Abcam PLC). Conditioned media from day 4 through day 28 was pooled together. Colorimetric absorbance was measured with a Tecan Infinite M200 PRO (Tecan) and analyzed with i-control 1.9 software (Tecan). Protein levels were then normalized to PBS injected discs from the same spine.

### Protein Arrays

Media collected from day 4 to day 28 was pooled together and analyzed use the RayBio Human Cytokine Antibody Array C6 (RayBiotech, Inc.) according to manufacturer’s instructions. Arrays were imaged using an ImageQuant LAS4000 Image Analyzer (GE) and analyzed with ImageQuant TL array analysis software (GE). Data was normalized to cytokine secretion from PBS injected discs from the same spine.

### Statistical Analysis

Data was analyzed with Graph Pad Prism Version 7. Data was analyzed by repeated-measures one-way ANOVA with Fisher’s least significant difference post-hoc text unless otherwise indicated, normalized to untreated cells of PBS injected discs, and presented at mean ± standard error of the mean (SEM).

### Data Availability

Data generated or analysed during this study are included in whole or in part in this published article. Where only part of a data set is included (*i.e*. mass spectrometry data), reasonable requests can be made to the corresponding author.

## Electronic supplementary material

Supplemental Information
