# Supplementary material for: Toll-like Receptor Activation Induces Degeneration of Human Intervertebral Discs
Source: Sci Rep. 2017 Dec 7;7:17184. doi: 10.1038/s41598-017-17472-1 (PMC5719358; doi:10.1038/s41598-017-17472-1)
Supplement: Supplementary file 1 — Supplemental Information [file 41598_2017_17472_MOESM1_ESM.pdf]

Toll-like Receptor Activation Induces Degeneration of Human Intervertebral Discs

Emerson Krock[a,b], Derek H. Rosenzweig[a,b], J. Brooke Currie[a,b], Daniel G Bisson[a,b], Jean A. Ouellet[b,c], *Lisbet Haglund[a-c]

a - Orthopaedic Research Lab and **b** McGill Scoliosis and Spine Group, Department of Surgery, McGill University and the Research Institute of the McGill University Health Centre, 1650 Cedar Ave, C10-148.8, Montreal, QC, Canada H3G 1A4

c - Shriner's Hospital for Children, 1003 Decarie Blvd, Montreal, QC, Canada H4A 0A9

***Corresponding Author's Address**:

Lisbet Haglund, Department of Experimental Surgery,
Montreal General Hospital, Research Institute of the McGill University Health Centre
Rm C10-148.2, 1650 Cedar Ave. Montreal, Qc, Canada H3G 1A4
Tel: (514)-934-1934 ext 35380
Fax: (514)-843-1699
E-mail: lisbet.haglund@mcgill.ca

Supplemental Figure 1

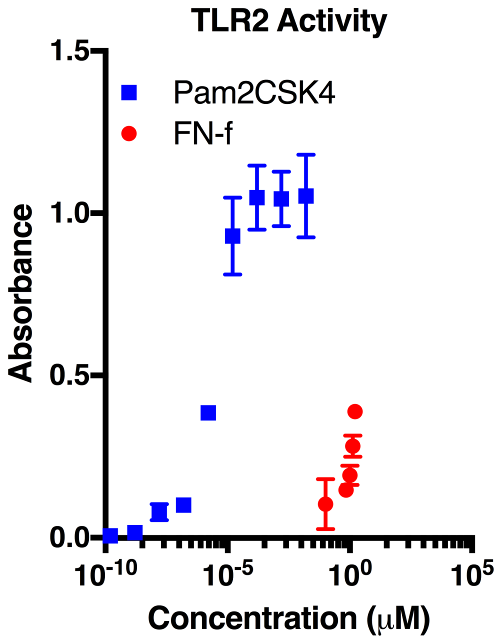

Supplemental Figure 1. TLR2 activity in response to Pam2CSK4 and 30kDa fibronectin fragments HEK 293 cells that over express TLR2 and produce SEAP following TLR2 activation were used to evaluate the activity patterns of Pam2CSK4 and 30 kDa fibronectin fragments (FN-f). TLR2 induction of SEAP activity causes the culture media to turn blue, increasing absorbance at 630nm. Cells were treated with 0.0002 pmol to 15.75 pmol Pam2CSK4 or 100 nmol to 1600 nmol FN-f for 12 hours and absorbance was measured. Data is presented as the mean ± SEM, n=4.

Supplemental Table 1

	Donor	1	2	3	4	5	6
	**Age**	57	50	42	48	46	44
**PBS**	Disc Level	L1/2	L3/4	L3/4	L2/3	L1/2	L3/4
	Grade based on Wilke *et al.*	1	1	0	1	1	1
	Disc Height (cm)	0.84	1.17	1.13	1.08	1.01	0.97
**Pam2CSK4**	Disc Level	L3/4	L2/3	L2/3	L3/4	L3/4	L1/2
	Grade based on Wilke *et al.*	1	1	0	1	1	1
	Disc Height (cm)	1.01	1.9	1.12	1.1	1.01	0.86
**FN-f**	Disc Level	L2/3	L1/2	L4/5	L4/5	L2/3	L4/5
	Grade based on Wilke *et al.*	1	1	1	1	1	0
	Disc Height (cm)	1.07	1.2	1.24	1.19	1.00	1.01

Supplemental Table 1. Characteristics of discs injected with each treatment. Age of an individual donor is specified in years. The level of the disc injected with either PBS, Pam2CSK4, and FN-f from the lumbar region is indicated. Discs were graded based on Wilke *et al.* 2006. Disc height was determined by averaging the dorsal, ventral and midsection disc height. No differences in grade or height were found between groups prior to treatment.

Supplemental Table 2. Primers used for RT-qPCR

Gene	Primer Sequence 5' to 3'	Primer Reference
GAPDH	F: TCCCTGAGCTGAACGGGAAG<br>R: GGAGGAGTGGGTGTCGCTGT	[46]
IL-1β	F: ACAGATGAAGTGCTCCTTCCA<br>R: GTCGGAGATTCGTAGCTGGAT	[47]
NGF	F: AAGTGCCGGGACCCAAAT<br>R: TGAGTTCCAGTGCTTTGAGTCAA	[48]
